# Regionale Charakteristika der Diagnosehäufigkeit unter im Ärztlichen Dienst der Bundesagentur für Arbeit begutachteten Arbeitslosen

**DOI:** 10.1055/a-2754-1400

**Published:** 2026-03-11

**Authors:** Andreas Guenter Franke, Norbert Scherbaum, Kirsi Marjaana Manz, Claudia Pieper, Gabriele Lotz-Metz

**Affiliations:** 1 Department of Counselling, University of Applied Labour Studies, Mannheim, Germany; 2 Ärztlicher Dienst, Bundesagentur für Arbeit, Nuremberg, Germany; 3 LVR-Universitätsklinik Essen, Klinik für Psychiatrie und Psychotherapie, Medizinische Fakultät der Universität Duisburg-Essen, Essen, Germany; 4 Klinik für Hämatologie, Hämostaseologie, Onkologie und Stammzellentransplantation, Medizinische Hochschule Hannover (MHH), Hanover, Germany; 5 Institut für Medizinische Informatik, Biometrie und Epidemiologie, Universitätsklinikum Essen, Essen, Germany

**Keywords:** Arbeitslosigkeit, Erkrankungen, Epidemiologie, Leistungsunfähigkeit, Regionen, Unemployment, illnesses, epidemiology, inability to work, regions

## Abstract

**Einleitung:**

Grundsätzlich besteht ein Zusammenhang zwischen Arbeitslosigkeit und der Gesundheit der von Arbeitslosigkeit Betroffenen. Für Deutschland fehlen für diesen Zusammenhang aktuelle Daten, insbesondere auf der Basis ärztlich gestellter Diagnosen und unter Berücksichtigung regionaler Besonderheiten.

**Methoden:**

Bundesweit wurden alle sozialmedizinischen Begutachtungen des Ärztlichen Dienstes der Bundesagentur für Arbeit zwischen 2016–2021 hinsichtlich Diagnosen, Beurteilung der Leistungsfähigkeit und regionaler Zuordnungen analysiert.

**Ergebnisse:**

Insgesamt wurden 4.249.028 Begutachtungen ausgewertet. Im Jahr 2021 wurden durchschnittlich 1,4% der erwerbsfähigen arbeitslosen Personen begutachtet, relativ am meisten in der Region Berlin-Brandenburg (1,9%), absolut am meisten in Nordrhein-Westfalen (19,2%). Relativ zur Arbeitslosenquote fanden die meisten Begutachtungen in Baden-Württemberg statt. In allen Regionen sind F-Diagnosen (psychische Erkrankungen) am häufigsten – vor allem bei den unter 25-Jährigen (3/4) und in Berlin-Brandenburg (62,4%). Am zweithäufigsten sind M-Diagnosen (muskulo-skelettale Erkrankungen) mit den höchsten Werten (35,9%) in Niedersachsen-Bremen und I-Diagnosen (Herz-/Kreislauferkrankungen) mit den höchsten Werten (14,8%) in Sachsen-Anhalt und Thüringen. Während die gesamte Krankheitslast in Nordrhein-Westfalen am höchsten liegt, hat Berlin-Brandenburg mit 40,7% die höchste Anzahl von leistungsunfähigen arbeitslosen Personen, wobei bundesweit insgesamt 33,9% aller begutachteten arbeitslosen Personen als leistungsunfähig (<3 Stunden arbeitsfähig/Tag) eingestuft wurden.

**Schlussfolgerung:**

Erkrankungen sind ein wichtiges Hindernis für das Durchbrechen von Arbeitslosigkeit. Entsprechend kann eine Behandlung helfen, in den Arbeitsmarkt zurückzukehren. Die hier aufgezeigten regionalen Varianzen weisen auf nur schwer erklärbare Unterschiede hin.

## Abstract (en)

**Introduction:**

There is a correlation between unemployment and health status of unemployed people. In Germany, there is a lack of current data on this relationship, particularly on the basis of medical diagnoses and regional characteristics.

**Methods:**

All socio-medical assessments by the Medical Service of the Federal Employment Agency between 2016–2021 were analyzed nationwide with regard to diagnoses, results of performance assessment and regional characteristics.

**Results:**

A total of 4,249,028 socio-medical assessments were analyzed. In 2021, an average of 1.4% of all unemployed were assessed, relatively most from the region of Berlin-Brandenburg (1.9%); in absolute terms, most in North Rhine-Westphalia (19.2%). Relative to the unemployment rate, most assessments took place in Baden-Württemberg. In all regions, F-diagnoses (mental disorders) led with the highest values among those who under the age of 25 (3/4) and living in Berlin-Brandenburg (62.4%), followed by M-diagnoses (musculoskeletal disorders) with the highest values (35.9%) in Lower Saxony-Bremen and I-diagnoses (circulatory disorders) with the highest values (14.8%) in Saxony-Anhalt and Thuringia. While the overall burden of disease was highest in North Rhine-Westphalia, Berlin-Brandenburg had the highest number of those being unable to work (40.7%). Overall, 33.9% of all assessed unemployed people were classified as being unable to work (<3 hours/day).

**Conclusion:**

Illnesses are an important obstacle to breaking through unemployment. Accordingly, treatment can help people to return to the labor market. The regional variations shown here indicate differences that are difficult to explain.

## Einleitung

Die Marienthalstudie hat in den 1930er Jahren gezeigt, dass Arbeitslosigkeit (ALO) mit körperlichen, seelischen sowie sozialen Beeinträchtigungen einhergeht 1 . Seither haben dies unzählige Studien bestätigt. Dies gilt beispielsweise für den Zusammenhang von ALO und Diabetes sowie Adipositas 2 3 , Krebserkrankungen 4 5 , Immobilität, Schmerzen, Fatigue (zum Beispiel bei muskuloskelettalen und rheumatoiden Erkrankungen) 6 7 8 9 10 sowie diverse psychische Erkrankungen 11 12 13 14 15 16 17 . Die Krankheitslast ist vor allem mit der Dauer der ALO verknüpft 15 16 17 18 19 .

Basierend auf der Marienthalstudie liegen auch für Deutschland zahlreiche (Studien-) Daten zum Zusammenhang von ALO und Erkrankungen vor. Diese Studien zeigen einen klaren Zusammenhang zwischen diversen Erkrankungen und (Langzeit-) ALO (zum Beispiel 19 20 21 22 23 24 ). Dabei weisen beispielsweise Übersichtsarbeiten von Henkel und KollegInnen sowie Nolte-Troha und KollegInnen, die auch auf Daten aus Deutschland basieren, auf den Zusammenhang von ALO und insbesondere Langzeitarbeitslosigkeit (LZA) mit Substanzkonsumstörungen hin 16 21 . Herbig und KollegInnen wiesen im Deutschen Ärzteblatt auf der Basis großer Meta-Analysen und systematischer Übersichtsarbeiten auf den Zusammenhang von LZA und psychischen Erkrankungen insbesondere bei Depressionen und Angststörungen hin 19 . Alfons Hollederer und KollegInnen wiesen unter anderem auf der Basis des German Labour Market and Social Security (PASS) Panels darauf hin, dass ALO unter anderem mit sogenannten „health-related characteristics“ assoziiert ist; dazu zählen unter arbeitslosen Personen das subjektive Gefühl, psychisch beeinträchtigt zu sein, an einer psychischen Krankheit zu leiden, häufigere Krankenhausaufenthalte und mehr Kontakte zu Ärzten 22 . Lampert und KollegInnen konnten mehrfach vor allem im Rahmen der GEDA-Studien (Gesundheit in Deutschland aktuell) und somit auf der Basis von Surveys zeigen, dass arbeitslose Personen im Vergleich zu Erwerbstätigen ihren subjektiven Gesundheitszustand deutlich schlechter einschätzten und häufiger unter ärztlich diagnostizierten Depressionen litten sowie deutlich weniger sportlich aktiv waren 23 25 26 . Die Arbeitsgruppe um Andreas G. Franke hat auf der Basis der Daten des Ärztlichen Dienstes (ÄD) der Bundesagentur für Arbeit (BA) gezeigt, dass ALO und vor allem LZA mit der Erkrankungshäufigkeit und der Leistungsfähigkeit der von ALO Betroffenen assoziiert ist; insbesondere psychische Erkrankungen sind unter von ALO Betroffenen besonders häufig 20 27 28 .

Hinsichtlich etwaiger topographischer Unterschiede der Bevölkerungsgesundheit geben die Studien zu den Global Burden of Disease nicht nur einen gesamtdeutschen Überblick, sondern geben auch Auskunft über bestimmte regionale Besonderheiten. In Deutschland sind im erwerbsfähigen Alter insgesamt ischämische Herzerkrankungen, Lungenkrebs, chronisch obstruktive Lungenerkrankungen, Diabetes mellitus sowie Schmerzen an Kopf und Rücken die häufigsten Gründe für gesundheitliche Einbußen und vorzeitige Sterblichkeit (disability adjusted life years, DALYs) 29 . Hinsichtlich der einzelnen Regionen innerhalb Deutschlands sind die DALY-Werte bezogen auf je 100.000 Einwohner in Baden-Württemberg und Teilen von Bayern am geringsten. Besonders hoch sind sie in Sachsen-Anhalt. Bei der chronisch obstruktiven Lungenerkrankung findet man die höchsten Werte an den westlichen Landesgrenzen mit Schwerpunkt Nordrhein-Westfalen und die niedrigsten in Baden-Württemberg und Bayern. Für Depressionen finden sich die niedrigsten Werte in den neuen Bundesländern (ohne Berlin) und die höchsten Werte in Berlin, Hamburg und einigen anderen Regionen 29 .

In Deutschland liegt die ALO-Quote bundesweit gegenwärtig bei rund 6,4% 30 . Von den rund 3 Mio. arbeitslosen Personen sind 1,9 Mio. gemäß Sozialgesetzbuch (SGB) II sogenannte langzeitarbeitslose Personen (ALO>1 Jahr), für 1,1 Mio. gelten die Regelungen des SGB III (ALO<1 Jahr). Am geringsten sind die ALO-Quoten in Bayern (4,1%) und Baden-Württemberg (4,5%), am höchsten in Bremen/Bremerhaven (11,7%), Berlin (10,2%), Mecklenburg-Vorpommern (8,4%), Nordrhein-Westfalen (7,9%), Sachsen-Anhalt (8,1%) und dem Saarland (7,5%).

Zuständig für die Vermittlung und weitere Dienstleistungen am Arbeitsmarkt ist die Bundesagentur für Arbeit mit rund 113 000 Beschäftigten.

Fraglich ist oft, ob von ALO betroffene Personen krank sind und deshalb nicht in den Arbeitsmarkt vermittelbar sind. Da den Vermittlungsfachkräften der Bundesagentur für Arbeit in der Regel eine ärztliche Expertise fehlt, bedarf es oft einer Einschaltung des Ärztlichen Dienstes der Bundesagentur für Arbeit. Die Vermittlungsfachkräfte entscheiden eigenständig, ob sie eine Notwendigkeit zur Einschaltung des Ärztlichen Dienstes sehen; diese liegt insbesondere dann vor, wenn die Vermittlungsfachkräfte das Beratungs-, Vermittlungs- oder Leistungsprocedere ohne eine fundierte ärztliche Stellungnahme nicht fortführen können.

Pro Jahr erhält der Ärztliche Dienst daher bundesweit rund 500 000 Begutachtungsaufträge 27 . Die sozialmedizinischen Begutachtungen werden dann bundesweit mit und ohne direkten KundInnenkontakt durchgeführt und basieren zudem auf der Auswertung medizinischer Unterlagen von BehandlerInnen der KundInnen, sofern die KundInnen der Einholung dieser Befunde zustimmen.

In Deutschland gibt es auch regionale Unterschiede hinsichtlich der Gesundheit von arbeitslosen Personen, die sich durch verschiedene Faktoren wie soziale Strukturen, wirtschaftliche Bedingungen, Zugang zu Gesundheitsversorgung und die regionalen Besonderheiten der Arbeitsmarktsituation und weitere Faktoren erklären lassen. Mehrere Studien vor allem des Robert-Koch-Instituts (RKI) (unter anderem RKI-Gesundheitsmonitoring, Gesundheitsberichterstattung) weisen beispielsweise auf unterschiedliche Lebenserwartungen und Krankheitsprävalenzen sowie unterschiedliches Gesundheitsveralten innerhalb Deutschlands hin, wobei vor allem Ost-West-Unterschiede deutlich werden/wurden (zum Beispiel 31 32 33 34 ). Arbeitslose Personen in den neuen Bundesländern sind häufig von schlechteren wirtschaftlichen Bedingungen betroffen, was sich negativ auf die Gesundheit auszuwirken scheint. Studien zeigen, dass in diesen Regionen häufig gesundheitliche Belastungen wie Depressionen, chronische Krankheiten und ein höheres Risiko für Frühverrentung aufgrund von gesundheitlichen Problemen vorliegen 35 . Zudem sind in den neuen Bundesländern Menschen mit Behinderungen signifikant weniger erwerbstätig als in den alten Bundesländern 36 . Ist in strukturschwachen Regionen, wie in ländlichen Gebieten oder in wirtschaftlich schwachen Städten, die höhere ALO-Quoten aufweisen, der Zugang zu Gesundheitsversorgung und sozialer Unterstützung eingeschränkt, wirkt sich dies wiederum negativ auf die Gesundheit von arbeitslosen Personen aus 37 . In größeren städtischen Ballungsräumen dagegen haben von ALO Betroffene tendenziell besseren Zugang zu medizinischer Versorgung, sozialen Programmen und psychologischer Unterstützung. Diese Regionen bieten oft mehr Ressourcen für Gesundheitsförderung und Prävention, was zu besseren Gesundheitsbedingungen für arbeitslose Personen führen kann.

Insgesamt haben arbeitslose Menschen, die in sozioökonomisch stärker deprivierten Regionen leben häufiger einen niedrigeren sozio-ökonomischen Status, sind häufiger von chronischen Erkrankungen und psychosomatischen Beschwerden betroffen, haben höhere Unfallverletzungswahrscheinlichkeiten, schätzen ihre eigene Gesund-heit schlechter ein und berichten häufiger von gesundheitsbeding -ten Einschränkungen 38 39 40 .

Bislang liegen für Deutschland keine Studien vor, die eine systematische Differenzierung zwischen ALO und Auftragsvergabe an den Ärztlichen Dienst sowie Krankheitslast und (berufliche) Leistungsfähigkeitsbestimmung der einzelnen Regionen systematisch untersuchen.

Ziel dieser explorativen Studie ist es daher, die Auftragsvergabe an den Ärztlichen Dienst, die Ergebnisse der Begutachtungen des Ärztlichen Dienstes (Diagnosen) und die berufliche Leistungsfähigkeit der KundInnen in einen regionalen Kontext zu setzen.

## Methoden

Es wurden Daten aller sozialmedizinischer Begutachtungen des Ärztlichen Dienstes der Bundesagentur für Arbeit zwischen 2016 – 2021 in der überregionalen Raumordnung des Ärztlichen Dienstes (Baden-Württemberg, Bayern, Berlin-Brandenburg, Hessen, Niedersachsen-Bremen, Nord (Mecklenburg-Vorpommern, Schleswig-Holstein, Hamburg), Nordrhein-Westfalen, Rheinland-Pfalz-Saarland, Sachsen, Sachsen-Anhalt-Thüringen) analysiert. Die Daten wurden auf einen entsprechenden Antrag der Forschenden/Autoren an die Stabsstelle Datenschutz aus den Datenbanken des Ärztlichen Dienstes (COMEDSQL, Microsoft SQL Server 2016) ausgelesen.

Grundlage der analysierten Daten sind die Ergebnisse der sozialmedizinischen Begutachtungen, die wiederum mit oder ohne KundInnenkontakt stattfanden. Sofern die KundInnen zur Vorbereitung auf die Begutachtung ihre behandelnden Ärzte von der Schweigepflicht gegenüber dem Ärztlichen Dienst entbanden, liegen dem Ärztlichen Dienst zur Begutachtung mehr oder weniger umfangreiche medizinische Befunde vor. Die eigentliche Begutachtung wird von erfahrenen ÄrztInnen des Ärztlichen Dienstes durchgeführt, von denen die überwiegende Mehrheit über eine Facharztqualifikation sowie die Zusatzqualifikation „Sozialmedizin“ verfügt 27 41 .

Die Datenbank COMEDSQL enthält demographische Daten der KundInnen wie Alter, Geschlecht, Region, Diagnosen gemäß der aktuellen Internationalen Klassifikation der Krankheiten (International Classification of Diseases, ICD-10) sowie sozialmedizinische Angaben über die Leistungsfähigkeit der KundInnen (<3 Stunden täglich, 3 – 6 Stunden täglich,>6 Stunden täglich).

Bei der Auswertung werden kontinuierliche Variablen mit Mittelwerten (MW) und Standardabweichungen (SD) angegeben; kategoriale Variablen werden mit Frequenzen und Prozentwerten angegeben. Diagnosen mit einer Häufigkeit von<1% werden unter „Sonstige“ zusammengefasst. Alle statistischen Berechnungen wurden mit „R“ (Version 4.3.2) durchgeführt.

Für diesen Beitrag wurden von den AutorInnen keine Studien an Menschen oder Tieren durchgeführt. Die Studie enthält keine personenbeziehbaren Daten und wurde unter Beachtung des Datenschutzes gemäß des Bundesbeauftragten für den Datenschutz und die Informationsfreiheit (BfDI) durchgeführt. Die Studie wurde seitens der Stabsstelle Datenschutz der Bundesagentur für Arbeit eingehend geprüft und im Oktober 2023 genehmigt (No. AZ1400.12-1/2023).

Es wurde keinerlei KI bei der Generierung der Daten oder der Erstellung des Manuskriptes genutzt.

## Ergebnisse

Die Datenbank des Ärztlichen Dienstes enthält für die Jahre 2016-2021 insgesamt rund 4,1 Mio. Datensätze zu Aufträgen an den Ärztlichen Dienst. Das mediane Alter der begutachteten Personen (sogenannte KundInnen) lag bei 45 Jahren (Min-Max 15-65 Jahre); 52,4% (n=2 147 620) waren männlich. In den einzelnen Regionen wichen die Werte davon etwas ab (siehe Tab. 1 ). Von den bundesweit insgesamt n=4 100 402 Aufträgen stammte die Mehrheit aus Nordrhein-Westfalen (19,2%, n=787 505), gefolgt von Baden-Württemberg (15,4%, n=631 642) und Bayern (12,9%, n=529 190) sowie der Region Berlin-Brandenburg (10,3%, n=422 711); die geringste Anzahl an Aufträgen stammte mit je 4,4% aus den Regionen Rheinland-Pfalz-Saarland (n=179 372) und Sachsen (n=181 345) (Details siehe Tab. 1 ).

**Table:** **Tab. 1** Verteilung der Aufträge nach Geschlecht, Alter und Region

Region	Gesamt (N=4 100 402)	Männliches Geschlecht (%, n)	Alter MW (SD)
Baden-Württemberg	15,4%, 631 642	53,8%, 339 589	43,7 (12,8)
Bayern	12,9%, 529 190	52,9%, 279 964	43,4 (13,2)
Berlin & Brandenburg	10,3%, 422 711	50,1%, 211 800	42,6 (13,1)
Hessen	7,7%, 317 775	52,9%, 168 110	43,9 (12,9)
Niedersachsen & Bremen	11,0%, 449 125	52,0%, 233 755	43,5 (13,3)
Nord (Mecklenburg-Vorpommern, Schleswig-Holstein, Hamburg)	9,4%, 383 917	49,9%, 191 598	42,7 (13,6)
Nordrhein-Westfalen	19,2%, 787 505	51,7%, 407 278	43,7 (13,0)
Rheinland-Pfalz & Saarland	4,4%, 179 372	52,8%, 94 644	43,6 (13,1)
Sachsen	4,4%, 181 345	55,0%, 99 751	42,0 (14,0)
Sachsen-Anhalt & Thüringen	5,3%, 217 820	55,6%, 121 131	44,0 (13,5)

Ursprung der Begutachtungsaufträge an den Ärztlichen Dienst in Anbetracht der Gesamtheit der Begutachtungsaufträge in den Jahren 2016 – 2021 (N=4 100 402) und das Geschlecht und Alter der begutachteten Arbeitslosen. MW=Mittelwert, SD=Standardabweichung.

In Tab. 2 werden die Aufträge des Jahres 2021 in Bezug zur jeweiligen Bevölkerung im erwerbsfähigen Alter und den Arbeitslosenzahlen gebracht. Demnach wurden im Jahr 2021 insgesamt 1,4% (734 348/53 193 690) der Bundesbürger im erwerbsfähigen Alter im Ärztlichen Dienst begutachtet. Die höchste relative Anzahl an Aufträgen zeigte sich in der Region Berlin-Brandenburg (1,9%, 73 969/3 991 212) gefolgt von der Region Nord (Mecklenburg-Vorpommern, Schleswig-Holstein, Hamburg) (1,7%, 70 990/4 070 847) und Niedersachsen-Bremen (1,6%, 86 893/5 533 647). Die geringste Beauftragungsquote war in der Region Rheinland-Pfalz-Saarland zu beobachten (1,1%, 34 120/3 230 784).

**Table:** **Tab. 2** Verteilung der Aufträge anhand der Bevölkerung und der Arbeitslosenzahlen im Jahr 2021

Region	Aufträge	Bevölkerung im erwerbsfähigen Alter (15 –<65 Jahre)	Ratio Aufträge/ Bevölkerung 15 –<65 Jahre (%)	Arbeits- Lose a) ges. b) SGB III c) SGB II	Ratio Aufträge/Arbeitslose (%) a) ges. b) SGB III c) SGB II
Gesamt	734 348	53 193 690	1,38	a) 2 613 489 b) 1 586 380 c) 1 027 109	a) 28,1 b) 46,3 c) 71,5
B-W	99 451	7 218 864	1,38	a) 247 774 b) 165 820 c) 81 954	a) 40,1 b) 60,0 c) 121,3
Bay	95 816	8 575 806	1,12	a) 262 186 b) 190 605 c) 71 582	a) 36,6 b) 50,3 c) 133,9
B-B	73 969	3 991 212	1,85	a) 276 863 b) 169 266 c) 107 598	a) 26,7 b) 43,7 c) 68,7
He	56 461	4 070 129	1,39	a) 178 086 b) 111 672 c) 66 414	a) 31,7 b) 50,6 c) 85,0
NHB	86 893	5 533 647	1,57	a) 282 313 b) 163 908 c) 118405	a) 30,8 b) 53,0 c) 73,4
Nord	70 990	4 070 847	1,74	a) 231 669 b) 143 882 c) 87 788	a) 30,6 b) 49,3 c) 80,9
NRW	141 257	11 523 746	1,23	a) 718 220 b) 389 302 c) 328 918	a) 19,7 b) 36,3 c) 42,9
RPS	34 120	3 230 784	1,06	a) 148 294 b) 92 822 c) 55 472	a) 23,0 b) 36,8 c) 61,5
Sa	36 192	2 413 972	1,50	a) 124 743 b) 73 506 c) 51 237	a) 29,0 b) 49,2 c) 70,6
S-Th	39 199	2 564 683	1,53	a) 143 342 b) 85 599 c) 57 743	a) 27,3 b) 45,8 c) 67,9

Tab. 2: Begutachtungsaufträge an den Ärztlichen Dienst aus dem Jahr 2021; Abkürzungen: Baden-Württemberg (B-W), Bayern (Bay), Berlin-Brandenburg (B-B), Hessen (He); Niedersachsen-Bremen (NHB), Nord (Mecklenburg-Vorpommern, Schleswig-Holstein, Hamburg), Nordrhein-Westfalen (NRW), Rheinland-Pfalz-Saarland (RPS), Sachsen (Sa), Sachsen-Anhalt-Thüringen (S-Th). In der Spalte „Ratio Aufträge/Arbeitslose“ können Prozentwerte>100% auftreten, wenn es in einer Gruppe mehr Aufträge als arbeitslose Personen gab. Das bedeutet, dass in diesen Gruppen mehrere Begutachtungen pro arbeitslose Person in Auftrag gegeben wurden; Quelle für Bevölkerungsdaten 2021 und Anzahl Arbeitslose 2021: Statistik der Bundesagentur für Arbeit; Zahlen, Daten, Fakten: Strukturdaten und -indikatoren; Deutschland, Hannover, Dezember 2022 https://statistik.arbeitsagentur.de/Statistikdaten/Detail/202212/iiia4/zdf-sdi/sdi-d-0-202212-xlsx.xlsx?__blob=publicationFile&v=1

Insgesamt wurden deutschlandweit im Jahr 2021 28,1% (734 348/2 613 489) der von ALO Betroffenen beim Ärztlichen Dienst vorgestellt. Bezieht man die Anzahl der Beauftragungen auf die Anzahl der Arbeitslosen je Region, so ergibt sich die höchste Beauftragungsquote in Baden-Württemberg (40,1%, 99 451/247 774), gefolgt von Bayern (36,6%, 95 816/262 186) und Hessen (31,7%, 56 461/178 086). Die niedrigsten Beauftragungsquoten finden sich in Nordrhein-Westfalen (19,7%, 141 257/718 220) und Rheinland-Pfalz-Saarland (23,0%, 34 120/148 294) (weitere Werte siehe Tab. 2 ).

Bundesweit sind die psychischen und Verhaltensstörungen (F-Diagnosen) die häufigsten vom Ärztlichen Dienst gestellten Diagnosen (54,0%, n=2 213 048), gefolgt von Krankheiten des Muskel-Skelett-Systems und des Bindegewebes (M-Diagnosen; 32,5%, n=1 334 633) und Krankheiten des Kreislaufsystems (I-Diagnosen; 11,1%, n=455 804) 20 . Von allen Diagnosen waren die F-Diagnosen in der Region Berlin-Brandenburg mit 62,4% (263 760/422 711) anteilig am häufigsten. Die wenigsten F-Diagnosen als Anteil an allen Diagnosen stammen mit 46,6% (101 591/217 820) aus Sachsen-Anhalt-Thüringen. Die entsprechenden Anteile bei den M-Diagnosen betragen 35,9% (161 371/449 125) für die Region Niedersachsen-Bremen (am häufigsten) und 28,7% (110 233/383 917) für die Region Nord (Mecklenburg-Vorpommern, Schleswig-Holstein, Hamburg) (am seltensten). Die I-Diagnosen sind in Sachsen-Anhalt-Thüringen mit 14,8% (32 212/217 820) am häufigsten und in Berlin-Brandenburg am seltensten (9,5%, 39 975/422 711). Weitere Angaben zur Diagnoseverteilung sind Tab. 3 zu entnehmen.

**Table:** **Tab. 3** Verteilung der beim Ärztlichen Dienst gestellten Diagnosen nach Regionen

	B-W	Bay	B-B	He	NHB	Nord	NRW	RPS	Sa	S-Th	Ges.
C00-D48 Neubildungen	4,9%, 30 939	4,2%, 22 087	3,5%, 14 708	4,7%, 15 087	4,2%, 18 814	4,3%, 16 417	4,7%, 37 357	4,4%, 7929	5,2%, 9385	5,0%, 10 941	4,5%, 183 664
E00-E90 Endokrine, Ernährungs- und Stoffwechselkrankheiten	6,5%, 40 889	6,2%, 32 886	5,8%, 24 635	7,2%, 22 931	6,4%, 28 893	6,8%, 26 013	6,8%, 53 840	8,3%, 14 824	7,3%, 13 152	7,4%, 16 148	6,7%, 274 211
F00-F99 Psychische und Verhaltensstörungen	51,2%, 323 537	55,6%, 294 405	62,4%, 263 760	51,3%, 162 881	51,6%, 231 924	57,7%, 221 434	54,5%, 429 150	49,0%, 87 909	53,2%, 96 457	46,6%, 101 591	54,0%, 2 213 048
G00-G99 Krankheiten des Nervensystems	7,9%, 50 151	7,8%, 41 150	6,7%, 28 472	7,7%, 24 334	7,4%, 33 373	7,2%, 27 687	7,5%, 59 028	7,6%, 13 593	7,7%, 13 915	8,1%, 17 718	7,5%, 309 421
I00-I99 Krankheiten des Kreislaufsystems	10,5%, 66 455	10,1%, 53 604	9,5%, 39 975	11,3%, 35 913	10,2%, 45 994	10,2%, 39 314	12,0%, 94 766	12,9%, 23 186	13,4%, 24 385	14,8%, 32 212	11,1%, 455 804
J00-J99 Krankheiten des Atmungssystems	2,0%, 12 359	2,0%, 10 568	2,1%, 9056	2,1%, 6793	2,3%, 10 342	2,1%, 8098	2,4%, 18 860	2,7%, 4866	2,0%, 3666	2,5%, 5426	2,2%, 90 034
K00-K93 Krankheiten des Verdauungssystems	2,8%, 17 962	2,9%, 15 115	2,4%, 10 295	2,9%, 9289	2,7%, 12 226	2,6%, 9901	2,4%, 18 776	2,8%, 5 048	3,3%, 5 960	3,1%, 6 855	2,7%, 111 427
M00-M99 Krankheiten des Muskel-Skelett-Systems und des Bindegewebes	33,7%, 212 880	33,5%, 177 111	28,1%, 118 795	33,5%, 106 359	35,9%, 161 371	28,7%, 110 233	33,2%, 261 126	33,1%, 59 318	30,7%, 55 632	33,0%, 71 808	32,5%, 1 334 633
R00-R99 Symptome und abnorme klinische und Laborbefunde, die anderenorts nicht klassifiziert sind	5,6%, 35 234	4,9%, 26 172	4,7%, 19 762	6,0%, 19 007	5,1%, 22 788	4,7%, 17 862	4,1%, 32 240	5,8%, 10 418	3,2%, 5808	4,3%, 9382	4,8%, 198 673
S00-T98 Verletzungen, Vergiftungen und bestimmte andere Folgen äußerer Ursachen	4,3%, 27 136	4,0%, 21 371	2,4%, 10 056	4,8%, 15 094	4,0%, 17 772	3,4%, 12 962	3,4%, 27 153	4,2%, 7566	3,8%, 6938	4,4%, 9603	3,8%, 155 651
Z00-Z99 Faktoren, die den Gesundheitszustand beeinflussen und zur Inanspruchnahme des Gesundheitswesens führen	5,6%, 35 328	4,5%, 23 826	3,2%, 13 482	5,0%, 15 956	4,6%, 20 511	3,8%, 14 671	3,3%, 25 877	5,6%, 10 016	3,8%, 6828	6,8%, 14 789	4,4%, 181 284
Sonstige	7,7%, 48 357	7,3%, 38 474	6,5%, 27 525	8,0%, 25 358	7,2%, 32 552	6,9%, 26 335	7,1%, 55 966	8,1%, 14 525	8,3%, 15 123	7,5%, 16 336	7,3%, 300 551
*Insgesamt*	*100%, 631 642*	*100%, 529 190*	*100%, 422 711*	*100%, 317 775*	*100%, 449 125*	*100%, 383 917*	*100%, 787 505*	*100%, 179 372*	*100%, 181 345*	*100%, 217 820*	*100%, 4 100 402*

Tab. 3: Verteilung der beim Ärztlichen Dienst gestellten Diagnosen nach Regionen auf der Basis der Beauftragung des Ärztlichen Dienstes zur Begutachtung in den Jahren 2016 – 2021; Abkürzungen: Baden-Württemberg (B-W), Bayern (Bay), Berlin-Brandenburg (B-B), Hessen (He), Niedersachsen-Bremen (NHB), Nord (Mecklenburg-Vorpommern, Schleswig-Holstein, Hamburg), Nordrhein-Westfalen (NRW), Rheinland-Pfalz-Saarland (RPS), Sachsen (Sa), Sachsen-Anhalt-Thüringen (S-Th), Ges.=Gesamt; Die Prozente wurden spaltenweise angegeben.

Betrachtet man die Teilgruppe der unter 25-jährigen begutachteten KundInnen, so zeigt sich, dass bei ihnen die F-Diagnosen mit ¾ aller Diagnosen anteilig deutlich häufiger vorkommen als bei älteren KundInnen (<25 Jahren: 75,3%, 95% Konfidenzintervall (KI): [75,1%, 75,4%] vs.≥25: 51,7%, KI: [51,6%, 51,7%], p<0,001). In der Teilgruppe der über 55-Jährigen sind die M-Diagnosen am häufigsten und treten deutlich häufiger auf als bei unter 55-jährigen KundInnen (≥55: 43,5%, KI: [43,4%, 43,6%] vs.<55: 30,9%, KI: [30,8%, 30,9%], p<0,001). Das mittlere Alter der KundInnen mit F-Diagnosen variiert zwischen 38,0 (SD: 14,1) Jahren in Sachsen und 41,1 (SD: 13.1) Jahren in Hessen bei einem deutschlandweiten Mittelwert von 40,6 (SD: 13,6) Jahren. Deutschlandweit sind 48,3% (1 069 574/2 213 048) der KundInnen mit F-Diagnosen männlich 20 . Dieser Anteil variiert zwischen 45,6% (100 993/221 434) in der Region Nord (Mecklenburg-Vorpommern, Schleswig-Holstein, Hamburg) und 51,1% (165 460/323 537) in Baden-Württemberg.

Viele KundInnen sind auf Grund der oben genannten Erkrankungen nicht oder nur eingeschränkt leistungsfähig 20 . Tab. 4 zeigt, dass insgesamt 33,9% (1 390 527/4100 402) der KundInnen nicht leistungsfähig sind (berufliche Leistungsfähigkeit<3 Stunden täglich), 4,4% (181 649/4100 402) sind eingeschränkt leistungsfähig (3 – 6 Stunden täglich), und 42,4% (1 736 931/4 100 402) der begutachteten KundInnen sind voll leistungsfähig (>6 Stunden täglich). Für 19,3% (791 295/4 100 402) der KundInnen wurde die Kategorie „keine Angabe“ angegeben, da im Datensatz keine expliziten Angaben verfügbar waren. Von den KundInnen mit einer expliziten Einschätzung des Grades der Leistungsfähigkeit, entfiel in Berlin-Brandenburg der höchste Anteil an allen begutachteten KundInnen, auf diejenigen, die als leistungsunfähig eingestuft wurden (40,7%, 172 108/422 711); der niedrigste Anteil an leistungsunfähigen Personen wurde mit 29,1% aus Rheinland-Pfalz-Saarland (52 131/179 372) und Sachsen (52 706/181 345) gemeldet.

**Table:** **Tab. 4** Leistungsfähigkeit der begutachteten KundInnen pro Region.

	Leistungsfähigkeit (Stunden am Tag)			
Region	<3 Stunden	3 – 6 Stunden	>6 Stunden	Keine Angabe
Baden-Württemberg	30,4%, 191 725	6,3%, 39 520	58,5%, 369 394	4,9%, 31 003
Bayern	32,4%, 171 229	6,2%, 32 803	41,5%, 219 574	20,0%, 105 584
Berlin & Brandenburg	40,7%, 172 108	4,7%, 19 917	22,6%, 95 732	31,9%, 134 954
Hessen	34,7%, 110 280	3,6%, 11 481	53,9%, 171 217	7,8%, 24 797
Niedersachsen & Bremen	32,8%, 147 424	2,4%, 10 636	39,3%, 176 533	25,5%, 114 532
Nord (Mecklenburg-Vorpommern, Schleswig-Holstein, Hamburg)	40,1%, 153 939	3,5%, 13 597	35,7%, 136 878	20,7%, 79 503
Nordrhein-Westfalen	34,5%, 271 590	4,1%, 32 362	38,8%, 305 895	22,6%, 177 658
Rheinland-Pfalz & Saarland	29,1%, 52 131	5,2%, 9373	42,9%, 76 954	22,8%, 40 914
Sachsen	29,1%, 52 706	3,4%, 6145	46,0%, 83 350	21,6%, 39 144
Sachsen-Anhalt &Thüringen	30,9%, 67 395	2,7%, 5815	46,6%, 101 404	19,8%, 43 206
*Insgesamt*	*33,9%, 1 390 527*	*4,4%, 181 649*	*42,4%, 1 736 931*	*19,3%, 791 295*

Tab. 4: Leistungsfähigkeit der begutachteten KundInnen pro Region auf der Basis der Beauftragungen des Ärztlichen Dienstes in den Jahren 2016 – 2021

## Diskussion

Ziel der explorativen Studie war es, einen Überblick über etwaige regionale Unterschiede von ALO und Krankheit, der damit assoziierten Leistungsfähigkeit sowie etwaiger regionaler Besonderheiten bei der Einschaltung des Ärztlichen Dienstes zu geben.

Ausgehend von der Tatsache, dass Erkrankungen mit Arbeitslosigkeit verknüpft sind und vor allem bei anhaltender Erkrankung die Wiederaufnahme einer beruflichen Tätigkeit schwieriger ist, könnte man vermuten, dass der Ärztliche Dienst in Regionen mit hoher ALO-Quote besonders häufig eingeschaltet wird (zum Beispiel Bremen/Bremerhaven, Berlin, Mecklenburg-Vorpommern, etc.) und in Regionen mit niedrigen ALO-Quoten signifikant seltener (zum Beispiel Bayern, Baden-Württemberg). Dabei darf aber nicht vergessen werden, dass Erkrankungen zu ALO führen sowie ALO wiederum die Entwicklung von Krankheiten begünstigt, so dass ein Teufelskreis beziehungsweise eine gleichsinnige Beziehung zwischen ALO und Erkrankungen entsteht (zum Beispiel 42 43 ).

Für den Untersuchungszeitraum von 2016 bis 2021 zeigen die Auswertungen die absolut höchsten Einschaltquoten des Ärztlichen Dienstes in Nordrhein-Westfalen (n=787 505); analog dazu verfügt Nordrhein-Westfalen über die bundesweit höchste Anzahl von Einwohnern (rund 18,1 Mio.) 44 sowie über die bundesweit höchste Anzahl an arbeitslosen Personen (aktuell 787 516), was zu einer ALO-Quote von 7,9% führt. Relativ zur Bevölkerungsanzahl hat die Region Berlin-Brandenburg die höchste Einschaltquote; dies korrespondiert mit der bundesweit zweithöchsten ALO-Quote in Berlin von 9,5%. Hinsichtlich der Region Berlin-Brandenburg und den übrigen Regionen lässt sich allerdings keine differenzierte Aussage treffen, da die Daten des Ärztlichen Dienstes bestimmte Regionen zusammenfassen (zum Beispiel Berlin-Brandenburg, Nord, Niedersachsen-Bremen), so dass Regionen mit hohen ALO-Quoten mit Regionen mit deutlich niedrigeren ALO-Quoten zusammengefasst sind (zum Beispiel Bremen und Niedersachsen). Bezüglich der Bundesländer mit den niedrigsten ALO-Quoten (Bayern: 3,5%, Baden-Württemberg: 4,1%) ist die Einschaltquote des Ärztlichen Dienstes lediglich in Bayern besonders niedrig, was mit der oben angeführten These korrespondiert. Bezieht man die Anzahl der Begutachtungsaufträge an den Ärztlichen Dienste aber nicht auf die Bevölkerungsanzahl, sondern auf die Anzahl der Arbeitslosen, so sticht Baden-Württemberg mit der höchsten Einschaltquote hervor, gefolgt von Bayern, obwohl Baden-Württemberg und Bayern die niedrigsten ALO-Quoten aufweisen. In Berlin-Brandenburg, der Region mit der zweithöchsten ALO-Quote, zeigen sich lediglich durchschnittliche Einschaltwerte des Ärztlichen Dienstes; die niedrigsten Einschaltquoten bezogen auf die ALO-Quote hat Nordrhein-Westfalen trotz einer sehr hohen Anzahl von arbeitslosen Personen (787 516) und einer recht hohen ALO-Quote von 7,9%. Es ergibt sich somit kein klarer Erklärungsansatz.

Bemerkenswert ist die hohe Einschaltquote des Ärztlichen Dienstes im Bereich des SGB II: Gerade Baden-Württemberg und Bayern als Bundesländer mit den geringsten Quoten für ALO sowie Langzeit-ALO haben die mit Abstand höchsten Einschaltquoten bei KundInnen aus dem SGB II-Bereich (4,34 und 4,01). Dies könnte darauf schließen lassen, dass sich die Vermittlungskräfte des Zusammenhanges zwischen Langzeit-ALO und Erkrankungen sehr bewusst sind und rasch arbeitsmarktpolitische Maßnahmen auf der Basis der stattgehabten Begutachtungsergebnisse veranlassen (wollen).

Da insbesondere Langzeit-ALO (SGB II, ALO>1 Jahr) mit Erkrankungen korreliert, ist hier eine sozialmedizinische Begutachtung von besonderer Bedeutung, um den Teufelskreis aus Dauer der ALO und (bestimmten) Erkrankung(en) nicht zu verfestigen oder gar zu verschlimmern 11 15 16 18 19 24 45 . Besonders niedrig ist die Quote von Einschaltung des Ärztlichen Dienstes zu Langzeit-ALO allerdings in Nordrhein-Westfalen mit seiner tendenziell eher höheren ALO-Quote. Diese Befunde könnten auf eine geringe Awareness des Zusammenhanges zwischen ALO und Erkrankungen bei den auftraggebenden Abteilungen des Ärztlichen Dientes (vor allem Arbeitsvermittlung) schließen lassen, und hohe (Langzeit-) ALO-Quoten könnten abgesehen von anderen wesentlicheren Faktoren unter anderem auch die Folge von späten Einschaltungen des Ärztlichen Dienstes und ein spätes arbeitsmarkpolitisches Eingreifen sein. Dies ist aber rein spekulativ.

Die Einschaltquoten des Ärztlichen Dienstes entsprechen im Bundesvergleich seiner personellen Ausstattung: So ist die Anzahl der ärztlichen Mitarbeitenden in Voll- und Teilzeit in Nordrhein-Westfalen am höchsten gefolgt von Bayern und Baden-Württemberg 27 . In den Regionen mit den niedrigsten Einschaltquoten (Rheinland-Pfalz-Saarland, Sachsen) ist auch die Ausstattung des Ärztlichen Dienstes mit ärztlichem Personal in Voll- und Teilzeit zumindest in Sachsen im bundesweiten Vergleich tendenziell gering 27 . Es ist im Zuge des Grundsatzes von Wirtschaftlichkeit, Sparsamkeit und Zweckmäßigkeit davon auszugehen, dass im Verlauf die personelle Ausstattung des Ärztlichen Dienstes an das Auftragsvolumen angepasst wurde und somit dem regionalen Bedarf entspricht, um eine bundesweite Gleichbehandlung der KundInnen zu gewährleisten.

Darüber hinaus ist die Bevölkerungsdichte einer betrachteten Region ein weiterer Einflussfaktor, da die ALO-Quote eine relative Größe, die Einschaltung des Ärztlichen Dienstes allerdings eine absolute Größe darstellt. Es gibt darüber hinaus mehrere weitere mögliche Erklärungsansätze für die unterschiedlichen Einschaltquoten: In einigen Bundesländern gibt es zum Beispiel stärker forcierte Maßnahmen zur Teilhabe am Arbeitsleben oder beruflicher Reintegration. In Regionen mit höherer ALO, wie zum Beispiel in Teilen von Mecklenburg-Vorpommern oder Sachsen-Anhalt, sind solche Maßnahmen vor allem aus topographischen Gründen schwieriger zugänglich, und die Arbeitsmarktintegration wird negativ von der Region des Wohnortes beeinflusst 36 . Davon abgesehen könnte auch eine verstärkte Sensibilisierung von (Vermittlungs-) Fachkräften der Arbeitsagenturen und Jobcenter zum Zusammenhang von ALO und Krankheitslast zu einer höheren Einschaltquote des Ärztlichen Dienstes gerade in Regionen mit einer höheren ALO-Quote führen. In Nordrhein-Westfalen gibt es beispielsweise immer wieder Projekte zur Förderung der Gesundheit und Integration von Arbeitssuchenden 46 47 .

Inwieweit unter den Auftraggebenden des Ärztlichen Dienstes die Kenntnis des oben genannten Zusammenhangs verbreitet ist und inwiefern diesbezüglich eine Sensibilität vorliegt und ob es diesbezüglich tatsächlich regionale Unterschiede gibt, kann auf Grund fehlender Daten nicht beurteilt werden.

Eine mögliche Erklärung für die hohen Einschaltquoten in Bayern und Baden-Württemberg können darüber hinaus weitere regionale Kontextfaktoren sein, die die negativen Auswirkungen von Arbeitslosigkeit auf die Gesundheit wiederum verstärken. Dadurch entstehen auch in Regionen mit vergleichsweise niedriger Arbeitslosigkeit erhöhte Einschaltquoten infolge einer stärkeren gesundheitlichen Belastung. Regionale Unterschiede im Gesundheitszustand von Arbeitslosen lassen sich zum Beispiel durch das Zusammenspiel struktureller, sozialer und kultureller Faktoren erklären. In wirtschaftsstarken Regionen mit niedriger Arbeitslosigkeit – etwa in Süddeutschland – kann Arbeitslosigkeit stärker stigmatisiert sein und zu erhöhtem psychosozialem Stress führen 48 49 . Gleichzeitig verschärfen höhere Lebenshaltungskosten und ein ausgeprägtes Leistungs- und Erwerbsethos finanzielle und emotionale Belastungen 50 . In Regionen mit hoher Arbeitslosigkeit wird Arbeitslosigkeit dagegen häufiger als „normal“ wahrgenommen, was die psychischen Folgen abmildern kann 51 . Darüber hinaus spielen Unterschiede in der medizinischen Versorgung und im Fallmanagement der Arbeitsagenturen eine Rolle 52 .

Bei den Begutachtungen wurden am häufigsten F-Diagnosen gefolgt von M-Diagnosen und I-Diagnosen gestellt 20 ; dies gilt für alle betrachteten Regionen. Die höchste Anzahl an F-Diagnosen entspricht den Ergebnissen der Mental Health Surveys der Weltgesundheitsorganisation, die angeben, dass circa 970 Mio. Menschen weltweit unter psychischen Erkrankungen leiden, was ungefähr 13% der weltweiten Bevölkerung entspricht 53 54 . In allen Regionen Deutschlands ist der Anteil der F-Diagnosen an allen Diagnosen unter begutachteten von ALO Betroffenen aber signifikant höher als in den Mental Health Surveys. Dies entspricht am ehesten dem bereits dargelegten hohen Grad des Zusammenhangs zwischen (Langzeit-) ALO und (psychischen) Erkrankungen. Die hier präsentierten regionalen Daten widersprechen aber den DALYs der Global Burden of Disease Studien sowie weiteren (inter-) nationalen Studien (zum Beispiel GEDA (Gesundheit in Deutschland aktuell)) hinsichtlich der Häufigkeitsverteilung (siehe oben), wobei Letztere größtenteils auf Befragungen und anderen (subjektiven) Quellen beruhen, ohne medizinische Expertise einzubeziehen. Zudem differenzieren die (inter-) nationalen Studien nicht hinsichtlich der Berufstätigkeit der Befragten. Die Daten der vorliegenden Studie entstammen der sozialmedizinischen Begutachtung durch (Fach-) Ärzte; außerdem stammen die Global Burden of Disease Daten aus 204 Ländern mit ganz unterschiedlichen Charakteristika (Demographie, unterschiedliche Gesundheits- und Sozialstaatssysteme, etc.), die mitunter kaum Ähnlichkeit zu den analysierten Regionen Deutschlands haben 55 . Allerdings enthalten die Daten der aktuellen Studie lediglich Diagnosen zu den sozialmedizinisch begutachteten KundInnen und basieren nicht auf der sozialmedizinischen Untersuchung aller arbeitsloser Personen oder einer entsprechend randomisierten Stichprobe, so dass die gesundheitliche Situation der nicht zur Begutachtung beim Ärztlichen Dienst vorgestellten KundInnen trotz des immensen Datenpools unklar bleibt.

Der Vergleich der Begutachtungsergebnisse widerspricht aber teilweise den regionalen DALYs in Deutschland, die Porst und Kollegen beschrieben haben 29 : jeweils bezogen auf je 100 000 Einwohner ist die Krankheitslast in Baden-Württemberg am geringsten, gefolgt von Bayern; besonders hoch sind die DALYs insgesamt in Sachsen-Anhalt, dem Landkreis Mecklenburgische Seenplatte, dem nördlichen Teil Niedersachsens, dem mittleren/westlichen Teil Nordrhein-Westfalens und dem Saarland 29 . Die gesamte Krankheitslast über alle Krankheiten/Diagnosen unserer Daten ist im Bundesland Nordrhein-Westfalen am höchsten, gefolgt von Baden-Württemberg und Bayern. Unabhängig von der Region sind dabei die F-Diagnosen führend.

Von ischämischen Herzerkrankungen ist gemäß Porst und Kollegen (DALYs in den Regionen Deutschlands) vor allem Sachsen-Anhalt, gefolgt von Vorpommern, den Regionen Thüringen und Sachsen sowie den Küstenregionen Schleswig-Holsteins und Niedersachsens betroffen; die niedrigsten Werte finden sich für Baden-Württemberg 29 . Die hier vorgelegten Daten zeigen die höchste Anzahl von I-Diagnosen bei ALO in den Regionen Sachsen und Sachsen-Anhalt-Thüringen sowie die niedrigste in Baden-Württemberg. Somit liegen erneut nur punktuell Entsprechungen vor, die vermutlich unter anderem darauf zurückzuführen sind, dass die Daten des Ärztlichen Dienstes eine etwas gröbere topographische Aufteilung haben als die übliche Einteilung in 16 Bundesländer; außerdem stellen ischämische Herzerkrankungen nur einen Teil der I-Diagnosen dar, so dass auch diese fehlende Entsprechung zu Verzerrungen führen dürfte. Für die Depressionen finden sich die niedrigsten DALY-Werte gemäß Porst und Kollegen in den neuen Bundesländern (mit Ausnahme Berlin) und die höchsten Werte in Berlin, Hamburg, Niederbayern Mittelfranken, der Grenze zu Belgien und den Niederlanden der Region um Arnsberg und dem Süd-Westen Baden-Württembergs 29 . Die höchste Anzahl an F-Diagnosen unserer Studie finden sich in Berlin-Brandenburg; dies entspricht der hohen DALY-Prävalenz der Depression in Berlin 29 . Dennoch gelten auch hier die bereits oben genannten Limitationen (Differenzierung der Regionen, Depressionen als nur ein Teil der F-Diagnosegruppe). Darüber hinaus stellen die Daten unserer Studie auf Grund der (Vor-) Auswahl der beim Ärztlichen Dienst vorgestellten KundInnen durch die Vermittlungs(fach)kräfte und andere Auftraggebende keine eigentlichen Prävalenzraten dar.

Die Studiendaten zeigen eine besonders hohe Anzahl von F-Diagnosen bei den unter 25-Jährigen. Dies entspricht dem ersten Häufigkeitsgipfel der Depressionen, dem frühen Beginn der Schizophrenien sowie den Angststörungen und Aufmerksamkeitsdefizit-/Hyperaktivitätsstörungen (ADHS), die zusammen einen signifikanten Anteil der psychiatrischen Krankheitslast vor allem in jungen Lebensjahren darstellen 56 .

F-Diagnosen stellen oft chronische oder chronisch-rezidivierende Erkrankungen dar 56 , was besonders besorgniserregend ist. Dass das SGB II mit entsprechender Langzeit-ALO einen hohen Anteil an F-Diagnosen hat, dürfte nicht überraschen, da psychische Erkrankungen signifikant mit der Dauer der ALO assoziiert sind 11 15 16 17 18 19 . Dies können die analysierten Daten aber nicht abbilden, da die Dauer der ALO nicht in den Daten enthalten ist.

Hinsichtlich der resultierenden Einbußen der Leistungsfähigkeit hat sich gezeigt, dass in Berlin-Brandenburg die höchsten Raten der Leistungsunfähigkeit bestehen (<3 Stunden täglich). Darüber hinaus ist der Anteil der F-Diagnosen in Berlin-Brandenburg am höchsten, und auch die Prävalenzen von Depressionen sind (neben anderen Regionen) gemäß Porst und Kollegen 29 am höchsten. Dabei führen psychische Erkrankungen häufiger als somatische Krankheiten zu einer prolongierten Arbeitsunfähigkeit oder gar vollständigen Leistungs- bzw. Erwerbsminderung (quantitatives Leistungsbild<3 Stunden täglich) 57 58 . Die Literatur zum Zusammenhang von ALO und Erwerbsminderungsrenten ist uneindeutig 59 60 61 : Die aktuellsten Studien zeigen auf Ebene der Bundesländer, dass ALO-Quoten mit der Prävalenz der Erwerbsminderungsrenten assoziiert sind 62 ; innerhalb von Bayern war die Anzahl der Frühverrentungen dort besonders hoch, wo hohe ALO-Quoten herrschten 63 . Besonders hohe Zugänge von Erwerbsminderungsrenten zeigen sich in Regionen mit hoher ALO-Quote und somit insgesamt in den neuen Bundesländern mit Betonung von Mecklenburg-Vorpommern und Berlin-Brandenburg 64 . Dies widerspricht tendenziell den hohen Erwerbsminderungsquoten bei psychischen Erkrankungen, bei denen die DALYs der Depressionen in den neuen Bundesländern signifikant geringer sind 29 , entspricht aber wiederum den tendenziell höheren DALYs von koronaren Herzkrankheiten auf dem Gebiet der neuen Bundesländer 29 . Eine klare Entsprechung zeichnet sich somit insgesamt nicht ab.

Aus den Ergebnissen der Studie sowie der Diskussion Letzterer kann man gewisse Handlungsempfehlungen ableiten: Die Auftraggebenden (vor allem Vermittlungsfachkräfte) des Ärztlichen Dienstes sollten bundesweit gleichermaßen hinsichtlich des Zusammenhangs zwischen ALO und gesundheitlichen Beeinträchtigungen und insbesondere hinsichtlich der dadurch bedingten Teilhabebeeinträchtigungen sensibilisiert werden, um eine bundesweite Gleichbehandlung der KundInnen zu gewährleisten. Abgesehen davon ist eine bundesweit einheitliche Kombination aus rascher ALO-Bekämpfung und Erkennung sowie Behandlung gesundheitlicher Einbußen wichtig, um eine Verfestigung der ALO und einer Chronifizierung von Erkrankungen vorzubeugen.

Aus wissenschaftlicher und gesundheitspolitischer Perspektive könnte die Etablierung eines langfristig angelegten Berichterstattungssystems über den Gesundheitszustand von Erwerbslosen zudem empfehlenswert sein.

### Limitationen

Die wesentliche methodische Stärke dieser Untersuchung ist die Größe der Stichprobe sowie die ärztliche Stellung der Diagnosen und die darauf basierende ärztliche Einschätzung der Leistungseinbußen im Gegensatz zu (inter-) nationalen Studien (zum Beispiel Gesundheit in Deutschland aktuell (GEDA)-Studien, Mental Health Survey), die größtenteils auf Befragungen von Betroffenen beruhen. Limitierend ist allerdings festzustellen, dass die Analyse auf „nur“ 0,5 Mio. KundInnendaten pro Jahr basiert, die beim Ärztlichen Dienst zur sozialmedizinischen Begutachtung vorgestellt wurden und nicht um die Daten aller registrierten von ALO Betroffenen (2,7 Mio.) oder einer randomisierten Stichprobe. Trotz der beeindruckenden Zahl von über 4 Mio. Begutachtungen, die in diese Analyse eingegangen sind, besteht keine Repräsentativität für die Gesamtheit der arbeitslosen Menschen in Deutschland, und es können somit keine Prävalenzdaten von Erkrankungen bei arbeitslosen Personen dargestellt werden. Schließlich entscheiden die Vermittlungsfachkräfte der Arbeitsagenturen, denen grundsätzlich eine (sozial-) medizinische Expertise fehlt, subjektiv bzw. eigenständig, ob es der Einschaltung des Ärztlichen Dienstes bedarf oder nicht. Somit wird an diesem Punkt eine nicht (sozial-) medizinisch fundierte (Vor-) Auswahl derjenigen getroffen, die beim Ärztlichen Dienst zur Begutachtung vorgestellt werden. Dies stellt einen nicht zu unterschätzenden Bias dar. Die Studienergebnisse erlauben es somit auf Grund des oben genannten Bias im eigentlichen Sinne nicht, von Krankheitsprävalenzen zu sprechen. Es gelten aber bundesweit bestimmte Standards bzw. Kriterien für die Einstellung und Beschäftigung von Vermittlungs(fach)kräften sowie für die Einschaltung des Ärztlichen Dienstes, die zentral festgelegt werden.

Weiterhin wird die Auswertung durch technische und datenschutzrechtliche Faktoren begrenzt, die keine Auswertung weiterer Variablen zulassen wie zum Beispiel Soziodemographie, Bildungsbiographie, Migrationshintergrund. Da diese Aspekte aber von wesentlicher Bedeutung sein dürften, wäre eine Auswertung solcher Daten von entscheidender Bedeutung.

## Ethikstatement

Für diesen Beitrag wurden von den Autorinnen und Autoren keine Studien an Menschen oder Tieren durchgeführt. Die Studie enthält keinerlei personenbeziehbare Daten und wurde unter Beachtung des Datenschutzes gemäß des Bundesbeauftragten für den Datenschutz und die Informationsfreiheit (BfDI) durchgeführt. Die Studie wurde seitens der Abteilung Datenschutz der Bundesagentur für Arbeit eingehend geprüft und im Oktober 2023 genehmigt (No. AZ1400.12-1/2023).

## Statement zur Verwendung von künstlicher Intelligenz

Es wurde keinerlei KI bei der Generierung der Daten oder der Erstellung des Manuskriptes genutzt.

## References

[1] Jahoda M Lazarsfeld P F Zeisel H Die Arbeitslosen von Marienthal. Ein soziographischer Versuch über die Wirkungen langandauernder Arbeitslosigkeit Frankfurt a.M. Suhrkamp Verlag 1975

[2] Varanka-Ruuska T Rautio N Lehtiniemi H The association of unemployment with glucose metabolism: a systematic review and meta-analysis Int J Public Health 2018 63 435 446 29170882 10.1007/s00038-017-1040-z

[3] Pedron S Emmert-Fees K Laxy M The impact of diabetes on labour market participation: a systematic review of results and methods BMC Public Health 2019 19 25 30616606 10.1186/s12889-018-6324-6 PMC6323654

[4] Chimienti M Morlino G Ingravalle F Unemployment Status Subsequent to Cancer Diagnosis and Therapies: A Systematic Review and Meta-Analysis Cancers (Basel) 2023 15 36900304 10.3390/cancers15051513 PMC10000747

[5] Koch R Wittekindt C Altendorf-Hofmann A Employment pathways and work-related issues in head and neck cancer survivors Head Neck 2015 37 585 593 24677561 10.1002/hed.23640

[6] O'Hara N N Isaac M Slobogean G P The socioeconomic impact of orthopaedic trauma: A systematic review and meta-analysis PLoS One 2020 15 e0227907 31940334 10.1371/journal.pone.0227907 PMC6961943

[7] Nwaru C A Nygard C H Virtanen P Musculoskeletal pain and re-employment among unemployed job seekers: a three-year follow-up study BMC Public Health 2016 16 531 27392125 10.1186/s12889-016-3200-0 PMC4938954

[8] Clay F J Newstead S V McClure R J A systematic review of early prognostic factors for return to work following acute orthopaedic trauma Injury 2010 41 787 803 20435304 10.1016/j.injury.2010.04.005

[9] Porter M E What is value in health care? N Engl J Med 2010 363 2477 2481 21142528 10.1056/NEJMp1011024

[10] Sandqvist G Scheja A Hesselstrand R Pain, fatigue and hand function closely correlated to work ability and employment status in systemic sclerosis Rheumatology (Oxford) 2010 49 1739 1746 20511345 10.1093/rheumatology/keq145

[11] Crespo-Facorro B Such P Nylander A G The burden of disease in early schizophrenia – a systematic literature review Curr Med Res Opin 2021 37 109 121 33095689 10.1080/03007995.2020.1841618

[12] Crowe L Butterworth P The role of financial hardship, mastery and social support in the association between employment status and depression: results from an Australian longitudinal cohort study BMJ Open 2016 6 e009834 10.1136/bmjopen-2015-009834 PMC4885313 27235296

[13] Lahelma E Unemployment and mental well-being: elaboration of the relationship Int J Health Serv 1992 22 261 274 1601545 10.2190/LKDE-1E0K-TANM-HQUY

[14] Lim G Y Tam W W Lu Y Prevalence of Depression in the Community from 30 Countries between 1994 and 2014 Sci Rep 2018 8 2861 29434331 10.1038/s41598-018-21243-x PMC5809481

[15] Limm H Heinmuller M Liel K Factors associated with differences in perceived health among German long-term unemployed BMC Public Health 2012 12 485 22738028 10.1186/1471-2458-12-485 PMC3403995

[16] Nolte-Troha C Roser P Henkel D Unemployment and Substance Use: An Updated Review of Studies from North America and EuropeHealthcare (Basel) 2023 11 10.3390/healthcare11081182 PMC10137824 37108016

[17] Virgolino A Costa J Santos O Lost in transition: a systematic review of the association between unemployment and mental health J Ment Health 2022 31 432 444 34983292 10.1080/09638237.2021.2022615

[18] Milner A Page A LaMontagne A D Long-term unemployment and suicide: a systematic review and meta-analysis PLoS One 2013 8 e51333 23341881 10.1371/journal.pone.0051333 PMC3547020

[19] Herbig B Dragano N Angerer P Health in the long-term unemployed Dtsch Arztebl Int 2013 110 413 419 23837086 10.3238/arztebl.2013.0413 PMC3702026

[20] Franke A G Roser P Scherbaum N Disease prevalence and working ability among unemployed people in Germany between 2016 – 2021: Analysis of a large dataset from the Federal Employment Public Health 2025 242 37 43 40022990 10.1016/j.puhe.2025.02.021

[21] Henkel D Unemployment and substance use: a review of the literature (1990-2010) Curr Drug Abuse Rev 2011 4 4 27 21466502 10.2174/1874473711104010004

[22] Hollederer A Voigtlander S [Health of the unemployed and its effects on labour market integration : Results of the Labour Market and Social Security (PASS) panel study, waves 3 to 7 (2008/09-2013)] Bundesgesundheitsblatt Gesundheitsforschung Gesundheitsschutz 2016 59 652 661 27090246 10.1007/s00103-016-2341-8

[23] Lampert T Kroll L E von der Lippe E Sozioökonomischer Status und Gesundheit Bundesgesundheitsblatt-Gesundheitsforschung-Gesundheitsschutz 2013 56 814 821 23703503 10.1007/s00103-013-1695-4

[24] Nolte-Troha C Neumann S Franke A G Der Teufelskreis zwischen Arbeitslosigkeit und Substanzgebrauchsstörungen Nervenheilkunde 2023 42 649 655

[25] Lampert T Kroll L E Müters S Messung des sozioökonomischen Status in der Studie „Gesundheit in Deutschland aktuell“ (GEDA) Bundesgesundheitsblatt-Gesundheitsforschung-Gesundheitsschutz 2013 56 131 143 23275948 10.1007/s00103-012-1583-3

[26] Lampert T Schmidtke C Borgmann L S The subjective health of adults in Germany J Health Monit 2018 3 61 68 35586373 10.17886/RKI-GBE-2018-073 PMC8848780

[27] Franke A G Manz K Lotz-Metz G Socio-medical assessment: an inventory of the Medical Service of the Federal Employment Agency Bundesgesundheitsblatt Gesundheitsforschung Gesundheitsschutz 2024 67 1031 1038 39026002 10.1007/s00103-024-03932-3

[28] Roser P Manz K Scherbaum N Prevalence of mental disorders and work ability among unemployed individuals in Germany: a register-based analysis of socio-medical assessments by the Federal Employment Agency between 2016 and 2021 BMC Public Health 2025 25 475 39910607 10.1186/s12889-025-21603-z PMC11800403

[29] Porst M Lippe E V Leddin J The Burden of Disease in Germany at the National and Regional Level Dtsch Arztebl Int 2022 119 785 792 36350160 10.3238/arztebl.m2022.0314 PMC9902892

[30] Bundesagentur für Arbeit (2025) Monatsbericht zum Arbeits- und Ausbildungsmarkt. Bundesagentur für Arbeit, Nürnberg.

[31] Lampert T Hoebel J Kuntz B Health inequalities among children and adolescents in Germany. Developments over time and trends from the KiGGS study J Health Monit 2019 4 15 37 35146241 10.25646/5871 PMC8822245

[32] Lampert T Muters S Kuntz B 30 years after the fall of the Berlin Wall: Regional health differences in Germany J Health Monit 2019 4 2 23 10.25646/6077 PMC8832371 35586335

[33] Prutz F Rommel A Utilization of inpatient medical care in Germany J Health Monit 2017 2 89 94 37168134 10.17886/RKI-GBE-2017-128 PMC10165898

[34] Prutz F Rommel A Thom J Utilisation of outpatient medical services in Germany – Results from GEDA 2019/2020-EHIS J Health Monit 2021 6 45 65 35146316 10.25646/8555 PMC8734077

[35] Kroll L E Schumann M Hoebel J Regionale Unterschiede in der Gesundheit – Entwicklung eines sozioökonomischen Deprivationsindex für Deutschland Journal of Health Monitoring 2017 2 103 12037168133

[36] Hollederer A Erwerbslosigkeit, Gesundheit und Präventionspotenziale Ergebnisse des Mikrozensus 2005 VS Verlag für Sozialwissenschaften 2011

[37] Kroll L E Lampert T Regionalisierung von Gesundheitsindikatoren Bundesgesundheitsblatt Gesundheitsforschung Gesundheitsschutz 2011 55 129 140 10.1007/s00103-011-1403-1 22286258

[38] Hoebel J Lampert T Subjective social status and health: Multidisciplinary explanations and methodological challenges J Health Psychol 2020 25 173 185 30230391 10.1177/1359105318800804

[39] Hoebel J Rommel A Schroder S L Socioeconomic Inequalities in Health and Perceived Unmet Needs for Healthcare among the Elderly in Germany Int J Environ Res Public Health 2017 14 28954436 10.3390/ijerph14101127 PMC5664628

[40] Lampert T Kroll L E Kuntz B Health inequalities in Germany and in international comparison: trends and developments over time J Health Monit 2018 3 1 24 35586261 10.17886/RKI-GBE-2018-036 PMC8864567

[41] Bundesärztekammer 2020 (Muster-) Kursbuch Sozialmedizin auf der Grundlage der (Muster-) Weiterbildungsordnung 2018. 1. Aufl Bundesärztekammer Berlin

[42] Hollederer A Psychische Gesundheit im Fall von Arbeitslosigkeit. Praktische Arbeitsmedizin 2008 12 29 32

[43] Pech E Freude G Zusammenhang zwischen eingeschränktem Gesundheitszustand und ArbeitslosigkeitDortmund: Bundesanstalt für Arbeitsschutz und Arbeitsmedizin 2010 1 9

[44] Landesbetrieb Information und Technik NRW (2024) Landesdatenbank Nordrhein-Westfalen. Düsseldorf. Res ource document:https://www.landesdatenbank.nrw.de/ldbnrw/online?operation = previous&levelindex=1&step=1&titel=Erge bnis&levelid = 1720601992002&acceptscookies=false#abreadcrumbZugegriffen: 28. August 2024

[45] Paljarvi T Martikainen P Leinonen T Non-employment histories of middle-aged men and women who died from alcohol-related causes: a longitudinal retrospective study PLoS One 2014 9 e98620 24874518 10.1371/journal.pone.0098620 PMC4038621

[46] Geuter G Dickersbach M Das Modellprojekt „Zentrum für Bewegungsförderung Nordrhein-Westfalen “Düsseldorf: Landesinstitut für Gesundheit und Arbeit des Landes Nordrhein-Westfalen (Hg.): Alltagsnahe Bewegungsförderung 2010 60Jg., S.17 18

[47] Haupt J Pieper C Gesundheitliche Belastungen bei Langzeitarbeitslosen im SGB II Bezug–Ergebnisse einer Teilnehmerbefragung der Evaluation der integrierten Gesundheits-und Arbeitsförderung für die Stadt Essen Das Gesundheitswesen 2016 78 78

[48] Paul K I Moser K Unemployment impairs mental health: metaanalyses J Vocat Behav 2009 74 264 282

[49] Wulfgramm M Life satisfaction effects of unemployment in Europe: The moderating influence of labour market policy Journal of European Social Policy 2014 24 258 272

[50] Jahoda M Wirkungen von Arbeitslosigkeit Wirtschaftspsychologie 1983 3 59 71

[51] Warr P Work, Unemployment, and Mental Health Oxford Clarendon Press 1987

[52] Dragano N Siegrist J Wahrendorf M Social inequalities in health and health behaviour: Evidence from a European perspective Public Health Reviews 2011 32 347 362

[53] Demyttenaere K Bruffaerts R Posada-Villa J Prevalence, severity, and unmet need for treatment of mental disorders in the World Health Organization World Mental Health Surveys JAMA 2004 291 2581 2590 15173149 10.1001/jama.291.21.2581

[54] World Health Organization 2022 World mental health report: transforming mental health for all.World Health Organisation Geneva

[55] The Lancet. The Global Burden of Disease Study 2019. Special Issue. Volume 396, Number 10258, p1129-1306; 2020.

[56] Tebartz van Elst L Schramm E Berger M Psychiatrie und Psychotherapie. Klinik und Therapie psychischer Erkrankungen Amsterdam Elsevier 2024

[57] Bundespsychotherapeutenkammer (2013) BPtK-Studie zur Arbeits- und Erwerbsunfähigkeit Psychische Erkrankungen und gesundheitsbedingte Frühverrentung. Bundespsychotherapeutenkammer, Berlin.

[58] Melzner L Kroger C Incapacity to work due to mental disorders-economic, individual, and treatment-specific aspects Bundesgesundheitsblatt Gesundheitsforschung Gesundheitsschutz 2024 67 751 759 38789543 10.1007/s00103-024-03894-6 PMC11230963

[59] Rische H Die Absicherung des Erwerbsminderungsrisikos – Handlungsbedarf und Reformoptionen RV aktuell 2010 1 1 9

[60] Mörschel R Rehfeld U Untersuchungen der Rentenzugänge im Zeitablauf, Teil I: Der Zugang an Berufs- und Erwerbsunfähigkeitsrenten in den Rentenversicherungen der Arbeiter und der Angestellten – ein Maß für Umfang und Entwicklung der Invalidität? Deutsche Rentenversicherung 1981 4 234 253

[61] Erlinghagen M Knuth M Unemployment as an institutional construct? Structural differences in non-employment between selected European countries and the United States Journal of Social Policy 2010 39 71 94

[62] Schubert M Behrens J Höhne A Erwerbsminderungsrenten wegen verschlossenem Arbeitsmarkt – der Arbeitsmarkt als Frühberentungsgrund Deutschen Rentenversicherung Bund (DRV-Schriften) 2007 55 237 254

[63] Ebert A Hoffmann H Steppich B 2007 Regionales Frühverrentungsgeschehen und AltersübergängeIn:Stecker Ch, Putzing M, Hinz P, Kaufmann I (Hrsg.): Smart Region. Projektergebnisse zum alternsgerechten Arbeiten in innovativen Regionen Band 70 DRV-Schriften BerlinS88 105

[64] Brussig M Erwerbsminderung und Arbeitsmarkt: Arbeitslosigkeit und regionale Unterschiede prägen Zugänge in ErwerbsminderungsrentenAltersübergangs-Report 2012 4 1 26

